# Gallbladder Cancer: Epidemiology and Prevention

**DOI:** 10.3390/cancers18193151

**Published:** 2026-09-29

**Authors:** Spyridon Peppas, Gabriel Amorim Moreira Alves, Gisella Figlioli, Andreas G. Tsantes, Rozeta Sokou, Georgios K. Nikolopoulos, Fares Ayoub, Stefanos Bonovas, Daniele Piovani

**Affiliations:** 1 Department of Gastroenterology and Hepatology, Baylor College of Medicine, Houston, TX 77030, USA; spyridon.peppas@bcm.edu (S.P.); fares.ayoub@bcm.edu (F.A.); 2 Department of Biomedical Sciences, Humanitas University, 20072 Pieve Emanuele, MI, Italy; gabriel.alves@st.hunimed.eu; 3 Clinical Epidemiology and Research Center (CERC), Department of Biomedical Sciences, Humanitas University, Via Rita Levi Montalcini 4, 20072 Pieve Emanuele, MI, Italystefanos.bonovas@hunimed.eu (S.B.); 4 IRCCS Humanitas Research Hospital, 20089 Rozzano, MI, Italy; 5 Microbiology Department, “Saint Savvas” Oncology Hospital, 11522 Athens, Greece; 6 Laboratory of Haematology and Blood Bank Unit, School of Medicine, “Attiko” Hospital, National and Kapodistrian University of Athens, 12462 Athens, Greece; 7 Neonatal Department, National and Kapodistrian University of Athens, Aretaieio Hospital, 11528 Athens, Greece; rosesok@med.uoa.gr; 8 Laboratory of Medical Statistics, Epidemiology and Public Health, Medical School, University of Cyprus, Nicosia 1678, Cyprus; nikolopoulos.georgios@ucy.ac.cy

**Keywords:** GBC, prevention, gallstones, obesity, physical activity, *Salmonella Typhi*, aflatoxin, risk stratification

## Abstract

Gallbladder cancer (GBC) is an uncommon but highly lethal malignancy, characterized by striking geographic clustering, late diagnosis, and a prevention profile that differs from other gastrointestinal malignancies. This narrative review synthesizes epidemiologic, mechanistic, clinical, and public-health evidence on GBC determinants with a prevention-oriented lens. Rather than merely cataloguing risk factors, we propose a pragmatic framework that combines epidemiologic credibility, population-attributable burden, baseline risk, biologic plausibility, feasibility, and the benefit-risk balance of possible interventions. The strongest opportunities for prevention arise from risk-stratified management of gallbladder disease and congenital biliary abnormalities, reduction of excess and dysfunctional adiposity, promotion of physical activity, control of chronic Salmonella carriage in endemic settings, and mitigation of selected food- and water-borne toxic exposures. Several other determinants, including diet, alcohol consumption, smoking, reproductive factors, and socioeconomic disadvantage, may inform risk stratification or broader cancer-prevention policy, but currently offer less specific actionability. Effective prevention will require matching interventions to context: population-wide metabolic and lifestyle prevention, targeted clinical management of high-risk gallbladder conditions, and endemic-area strategies addressing infection, environmental exposure, and access to surgery. GBC prevention is therefore possible through carefully evaluated risk-adapted strategies.

## Simple Summary

Gallbladder cancer is a relatively rare cancer but is very often diagnosed at an advanced stage and has a poor prognosis. Its occurrence varies markedly across the world, with particularly high rates in parts of South America and Asia. This review examines the main factors associated with gallbladder cancer and considers how they may be used for prevention. Important opportunities include appropriate management of gallstones and other high-risk gallbladder conditions, prevention of excess body fat and physical inactivity, control of chronic Salmonella infection in endemic regions, and reduction of exposure to contaminants such as aflatoxins and heavy metals. However, the strength of the evidence and the feasibility of prevention differ substantially across risk factors. Prevention strategies should therefore be adapted to the baseline risk of different populations and to the expected benefits and harms of each intervention.

## 1. Introduction

Gallbladder cancer (GBC) is an uncommon but highly lethal malignancy [1]. The predominant histologic type is adenocarcinoma, and a major carcinogenic pathway involves chronic inflammation and progression through metaplasia, dysplasia, and carcinoma [2,3,4,5]. Early detection is uncommon because symptoms are non-specific and many tumors present at an advanced, unresectable stage [6]. Surgical resection remains the only potentially curative treatment, but only a minority of patients are diagnosed at a stage amenable to curative surgery [6]. Incidence varies markedly by region and population. High-risk areas include countries along the Andean region of South America, northern India and neighboring South Asian regions, East Asia, and selected Indigenous or ancestry-defined populations, whereas rates are much lower in many Western countries [1,2,7,8,9,10,11]. This geographic heterogeneity indicates that baseline risk is not fixed, but reflects the local distribution of risk factors such as gallstones, sex and reproductive patterns, metabolic risk, chronic infections, environmental exposures, ancestry, socioeconomic position, and access to timely biliary surgery [2,7,8,10,11].

The prevention challenge is correspondingly heterogeneous. Some determinants are anatomically proximate and clinically actionable, including gallstone disease, gallbladder polyps, primary sclerosing cholangitis, pancreaticobiliary maljunction, and choledochal cysts; others are systemic, behavioral, infectious, environmental, or structural, including adiposity, physical inactivity, *Salmonella Typhi/Paratyphi* carriage, aflatoxin exposure, drinking-water contamination, tobacco, alcohol, and educational disadvantage [7,8]. These determinants differ widely in evidence strength, population-attributable burden, feasibility of intervention, and acceptable benefit–risk thresholds.

This narrative review synthesizes epidemiologic, mechanistic, clinical, and public-health evidence on GBC determinants with emphasis on prevention. For each domain, we consider the credibility and magnitude of the association, biologic plausibility, population relevance, and clinical or public-health actionability. The review integrates evidence on potentially modifiable risk factors with gallbladder-specific clinical conditions, guideline recommendations, and regional context to examine how individual determinants may inform primary prevention, risk stratification, surveillance, or early detection. Epidemiological association, etiologic credibility, population-attributable burden, and evidence of intervention efficacy are considered as distinct dimensions rather than interchangeable indicators of preventability. Throughout the review, we distinguish primary prevention, aimed at reducing the occurrence of GBC, from risk-based surveillance of individuals at increased risk and early detection of an already developing malignancy. Surveillance and early-detection strategies are discussed where they are integral to the management of high-risk gallbladder conditions, but should not be interpreted as primary prevention.

### Review Approach

This narrative review did not aim to reproduce a de novo systematic review of all GBC risk factors. For environmental and potentially modifiable determinants, the principal epidemiological evidence base was a recent field-wide systematic review and meta-analysis that evaluated associations with GBC and graded their credibility [7]; population-attributable burden estimates were derived from a subsequent modelling analysis [8]. To address prevention, clinical actionability, surveillance, and management of high-risk gallbladder conditions, PubMed and Embase were additionally searched up to March 2026 using combinations of terms related to GBC and the relevant determinant, together with terms for prevention, screening, surveillance, cholecystectomy, and clinical management. Major clinical guidelines were also identified and critically compared. Priority was given to systematic reviews and meta-analyses, large prospective studies, GBC-specific evidence, and major society guidelines; primary studies were used where higher-level evidence was unavailable or where they addressed specific clinical or mechanistic questions.

For environmental determinants, the terms high, moderate, and low epidemiologic credibility follow the prespecified criteria of the field-wide systematic review [7], based on longitudinal meta-analyses and incorporating statistical significance, number of GBC cases, heterogeneity, small-study effects, and robustness to unmeasured confounding. Case–control evidence was considered complementary and was not formally graded. Mendelian randomization evidence and population-attributable fraction (PAF) estimates were considered separately: the former as additional evidence relevant to causal inference and the latter as a model-based measure of population impact conditional on causal assumptions.

## 2. Epidemiology

### 2.1. Incidence, Mortality, and Geographic Clustering

GBC shows one of the most uneven geographic distributions among gastrointestinal cancers. Global cancer estimates and GBD data indicate high burden in parts of South America, South Asia, and East Asia, with marked variation across countries and within regions [1,2,9,10,11]. According to GLOBOCAN 2022, there were 122,491 new cases worldwide, corresponding to an age-standardized incidence rate of 1.2 per 100,000, and 89,055 deaths, corresponding to an age-standardized mortality rate of 0.83 per 100,000 [1]. The absolute burden was concentrated in Asia: China, India, and Japan had the largest numbers of incident cases, with 31,132, 21,870, and 10,814 cases, respectively, and also the largest numbers of deaths, with 24,543, 16,407, and 7252 deaths, respectively [1].

**Figure 1:**
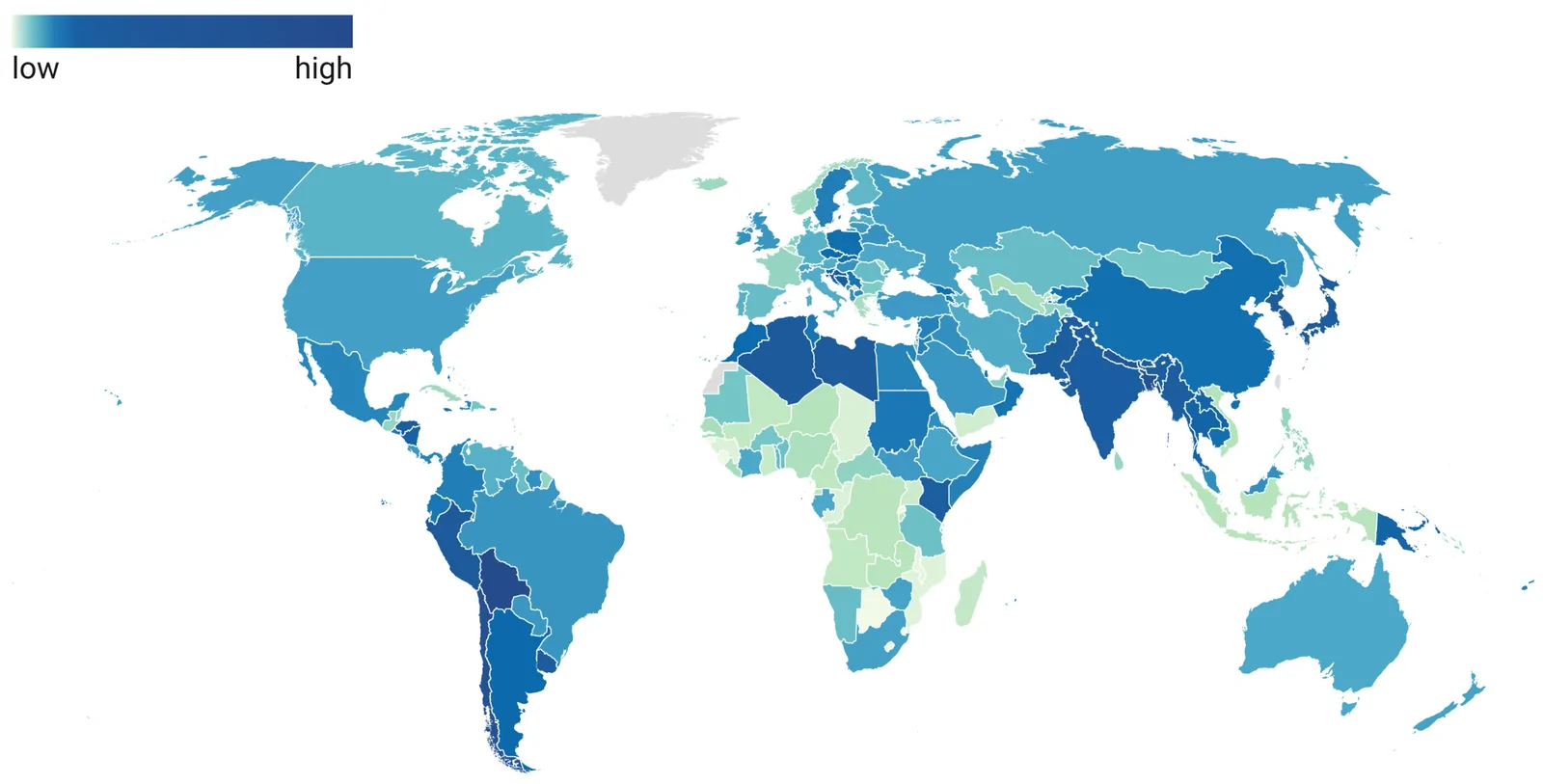
Global distribution of age-standardized incidence rates (ASR) of gallbladder cancer in 2022. Country-specific ASRs (per 100,000 person-years) were obtained from the GLOBOCAN 2022 database. The choropleth map was created using the online tool datawrapper.de (https://www.datawrapper.de/ accessed on 16 July 2026). Countries were grouped into deciles according to their ASR values, with map colors representing these decile categories; areas without available estimates are shown in grey.

The highest age-standardized incidence rates occur in more geographically restricted high-risk populations. In 2022, the highest country-level rates were reported in Bolivia, Chile, Bangladesh, Nepal, and Algeria, ranging from 2.7 to 7.6 cases per 100,000, compared with the global rate of 1.2 per 100,000 [1]. Mortality showed a similar pattern, with Bolivia reporting the highest age-standardized mortality rate, at 6.3 deaths per 100,000 [1]. GBC also shows a consistent female predominance: globally, women accounted for 78,953 incident cases and 57,649 deaths, compared with 43,358 cases and 31,406 deaths in men [1]. Among women, the highest incidence rates were reported in Bolivia, Bangladesh, Chile, Nepal, and Peru, ranging from 3.8 to 8.4 cases per 100,000 [1]. Figure 1 presents a global map of age-standardized GBC incidence rates using country-level GLOBOCAN 2022 estimates [1].

### 2.2. High-Risk Populations

GBC risk increases with age and is consistently higher in women, a pattern that broadly parallels the epidemiology of gallstone disease and reproductive factors, but age and sex do not by themselves explain the marked geographic concentration of the disease [1,2,10]. High-risk populations are better understood as groups in which demographic susceptibility, ancestry, gallbladder disease, environmental exposure, infection, and access to biliary surgery cluster in different combinations.

Ancestry-defined differences provide an important clue, particularly in the Americas. High rates have long been described in Andean and Indigenous populations, and population-based US data show disproportionate incidence among American Indian and Alaska Native populations [2,12]. In Chile, studies separating Mapuche and Aymara components of Native American ancestry showed that Mapuche ancestry, rather than broad Native American ancestry alone, was specifically associated with GBC mortality and risk [13]. Instrumental-variable analyses using ancestry-informative markers further suggested that the association between Mapuche ancestry and GBC is at least partly mediated by gallstone disease, while genetic work in admixed Chilean Latinos identified variants in ABCG8 and TRAF3 associated with gallstone disease and GBC [14,15].

Exposure-related explanations are also likely to vary by region. In northern India and neighboring South Asian regions, high incidence has been discussed in relation to gallstone burden, female predominance, reproductive patterns, chronic biliary infection, water and environmental exposures, and socioeconomic or health-system factors [7,11]. In South America, the available literature points to an overlapping but distinct profile, with gallstones, ancestry, reproductive history, environmental exposures, and access to cholecystectomy all considered relevant [2,10,13,14]. In East Asia, particularly Japan, a distinct component of GBC risk is represented by congenital biliary abnormalities such as pancreaticobiliary maljunction and congenital biliary dilatation, which have informed greater use of imaging-based identification and risk-adapted surgical management [16,17,18,19]. Thus, the relative contribution of gallstone disease, infection, environmental exposure, congenital abnormalities, and access to biliary care differs substantially across high-incidence regions.

## 3. Determinants of GBC and Prevention Implications

### 3.1. Gallbladder Diseases and Congenital Abnormalities

#### 3.1.1. Gallstones

Cholelithiasis is one of the strongest risk factors for the development of GBC, with 70–90% of cases having history of gallstone disease [6]. Despite the described link of gallstone disease with GBC, only 0.5–3.0% of patients with cholelithiasis develop GBC, suggesting that other factors are involved in its pathogenesis.

The most robust evidence on the association between gallstone and GBC originates from a large meta-analysis of 55 observational studies conducted in Europe, America and Asia including >13 million participants [20]. Among 22 studies, gallstone disease was associated with a pooled relative risk (RR) of 3.81 for GBC (95% CI: 3.02–4.82) [20]. Similarly, in an earlier pooled analysis, Randi et al. synthesized data from cancer registry-based and case–control studies across multiple continents, reporting a pooled RR of 4.9 (95% CI: 3.3–7.4), with the highest GBC incidence rates observed in women from India, Pakistan, and Ecuador [10]. However, the authors acknowledged the limitation of the observational design of included studies to establish causal relationships. This gap was aimed to be addressed by Mendelian Randomization (MR) studies [21,22], with an analysis of European and Chilean population showing a causal effect in both populations (Chileans odds ratio [OR] 1.97, *p* < 0.0001, and Europeans OR 5.02, *p* < 0.0001, respectively) [22]. In some cases, the risk estimates derived from MR studies were significantly smaller than those from epidemiologic studies, suggesting that residual confounding tend to overestimate the observed association in observational studies [21,22,23].

The risk of GBC is not uniform across patients with gallstones, and other factors play a determinant role on this association such the size, weight, volume and type of stones [24]. Diehl et al. performed a case–control study, which matched 81 GBC cases with controls for age, sex, hospital, and admission date, demonstrated that stones ≥3 cm conferred an OR of 10.1 (95% CI: 2.6–39.7) for development of GBC [25]. Similarly, a prospective trial investigating the role of size and number of gallstones in the development of GBC, demonstrated that patients with gallbladder carcinoma had significantly larger stones, a finding that was independent of the number of stones present (*p* < 0.001) [26]. This finding was subsequently replicated in an interracial study of 1676 female cholecystectomy patients across three racial groups, suggesting that stone size may serve as a surrogate marker for duration of gallstone disease and cumulative mucosal exposure. Although a relationship between the size and GBC has been observed in many retrospective studies [25,26,27,28], other studies did not reach the same observation [29,30]. A case–control study of 125 patients (93 with GBC and 31 with gallbladder dysplasia) matched to controls with chronic cholecystitis showed significantly higher mean weight (9.6 g vs. 6.0 g, *p* < 0.001) and volume (11.8 mL vs. 6.8 mL, *p* = 0.002) of gallstones in the GBC group compared to controls [30]. These findings suggest that adjunctive factors to the simple presence of gallstones may help guide in risk stratification and establishment of screening policies in high-risk groups.

The pathophysiological basis supporting the association of gallstones with GBC is driven by the theory of chronic inflammation and mucosal irritation [2]. It is postulated that the injury caused by the chronic presence of gallstones activates the inflammatory cascade with various mediators including chemokines, reactive oxygen species and prostaglandins, which induce mutations in oncogenes and tumor suppressor genes, DNA methylation and defects in base impair excision pathways [3]. These molecular insults lead to progressive accumulation of mutations in tumor suppressor genes (TP53, CDKN2A) and epigenetic alterations, driving a well-characterized histologic progression from metaplasia through dysplasia to adenocarcinoma, as confirmed by epigenome-wide methylation profiling demonstrating three distinct carcinogenic stages [4,5].

Despite the strong association of gallstone disease and GBC, the low GBC absolute risk in gallstone patients does not necessitate prophylactic cholecystectomy in most populations [31]. However, in high incidence countries, such as Chile and India, national screening policies have been implemented [32]. Chile established a prevention program in 2005, with prophylactic cholecystectomy in women aged 35–49 years old with gallstones, which resulted in greater GBC mortality decline in this patient group, supporting the hypothesis that prioritization of surgery in young females has contributed to that [33]. A study from India showed that 67 cholecystectomies could prevent a case of GBC, while in Chile, and after incorporating a risk prediction model, the number needed to treat (NNT) was 80 [32,34]. These estimates illustrate that the potential benefit of prophylactic cholecystectomy is highly dependent on baseline GBC risk and the ability to identify appropriately selected patients. In most populations, the low absolute risk of GBC among individuals with gallstones does not justify routine prophylactic surgery. Decisions should therefore incorporate individual and regional baseline risk, operative morbidity, competing risks, healthcare-system capacity, and cost-effectiveness.

#### 3.1.2. Porcelain Gallbladder and Chronic Inflammation

Gallbladder calcification, defined as “porcelain gallbladder”, has been historically accepted as an indication for cholecystectomy given its association with GBC [35]. However, recent studies have reported that the true incidence rate of GBC in patients with porcelain gallbladder is lower than initially expected, questioning the role of routine prophylactic cholecystectomy in this patient group [35].

The largest systematic review to date, including 111 studies (case series and cross-sectional studies) with 340 patients with gallbladder calcifications, found a 21% overall prevalence of GBC [36]. However, due to concerns regarding selection bias, the authors conducted a subgroup analysis of higher-quality studies in which selected populations more closely resembled those of the general population. In this higher quality subgroup, GBC was diagnosed in approximately 6% of patients with porcelain gallbladder compared to 1% in matched controls (RR 8.0, 95% CI: 1.0–63.0) [36]. A retrospective review of 10,741 cholecystectomies at UCLA (University of California, Los Angeles) Medical Center from 1955 to 1998 demonstrated gallbladder calcifications in 15 (0.14%) of surgical specimens with no evidence of carcinoma [37]. Likewise, in two large cohort studies the incidence of porcelain gallbladder was low (0.17–0.2%), with the presence of GBC in 5% and 15%, respectively [38,39].

Porcelain gallbladder is thought to result from dystrophic calcification secondary to chronic cholecystitis, often in the setting of gallstone disease which is present in 60–100% of patients with porcelain gallbladder [39]. Chronic irritation of the gallbladder wall caused by gallstones and cystic duct obstruction with bile stasis is thought to lead to mucosal calcium precipitation [35,40]. It is worth mentioning that the distribution and pattern of calcifications may play a role in the risk of GBC, as prior retrospective studies have demonstrated that diffuse calcification is associated with lower risk for GBC compared to minimal or no calcifications [41,42]. Thus, the carcinogenic pathway likely involves chronic inflammation and regeneration rather than calcification per se.

Traditionally, prophylactic cholecystectomy was recommended for all cases of porcelain gallbladder [36]. However, evidence from large retrospective studies has challenged this approach [35,36]. Current practice suggests prophylactic cholecystectomy for otherwise healthy patients because of the poor prognosis associated with GBC, while observation may be considered in elderly patients or those with significant comorbidities, as a cohort study found no significant difference in adverse events between observation and surgical management, and no patients developed GBC during follow-up [43]. Thus, a more nuanced approach should incorporate factors such as symptoms suggestive of malignancy, imaging findings, the pattern of calcification, and the presence of other risk factors, all of which can help guide the decision regarding cholecystectomy.

#### 3.1.3. Gallbladder Polyps

Gallbladder polyps are mainly diagnosed incidentally on imaging in asymptomatic individuals and are found in 4–7% of patients with GBC [44]. Given the increasing use of imaging in daily practice, the incidence of gallbladder polyps has been rising over the last years [44]. Most gallbladder polyps are non-neoplastic (i.e., cholesterol polyps, inflammatory polyps); however, polyps that are true adenomas and of size ≥10 mm carry a malignant potential, with current society guidelines recommending cholecystectomy [45]. Adenomyomatosis is a benign, non-neoplastic proliferative disorder of the gallbladder wall that may mimic polypoid or wall-thickening lesions. Although adenomyomatosis is not considered an established premalignant lesion, observational cholecystectomy series have reported a higher prevalence of GBC, particularly with the segmental type [46,47,48,49]. Rare carcinomas arising within Rokitansky–Aschoff sinuses have also been described [48]. Clinically, segmental or diffuse adenomyomatosis may complicate the radiological detection of coexisting early GBC because of gallbladder wall thickening; magnetic resonance cholangiopancreatography (MRCP) may be useful when ultrasound findings remain indeterminate [46,47,48,49].

There is a known association between gallbladder polyps and GBC, but the true malignant potential is low given that a minority of them are adenomas [50,51]. In a systematic review of 12 observational studies including a total of 5482 gallbladder polyps, the incidence of malignant and true adenomatous polyps was just 0.57% and 0.60%, respectively [52]. Among 64 patients diagnosed with a malignant gallbladder polyp, only one patient was found to have polyp size < 6 mm [52]. Additionally, the authors of the review concluded that established risk factors from included studies were: (i) size greater than 6 mm, with risk increasing with size, (ii) growth of polyp during follow-up, (iii) single polyp, and (iv) Indian ethnic background [52]. The main limitation of this study was the variability in the reporting of polyp characteristics within studies, along with the retrospective nature of some of the included studies which used imaging reports, and not the actual images.

Although gallbladder polyp size remains the strongest predictor of malignancy, there is a debate on the ideal threshold, and whether other factors truly add prognostic value. In the 2022 European guidelines, a cut-off of 10 mm for cholecystectomy, age > 60, primary sclerosing cholangitis (PSC), Asian ethnicity and sessile morphology were cited as risk factors, also recommending reassessment when polyps grow by ≥2 mm [45]. However, in a cohort of 438 patients, only size was predictive of cancer (*p* < 0.001), with age, symptoms, number of polyps, and growth all failing to reach significance [53]. Additionally, authors showed that size of 21 mm was cut-off for risk of cancer (area under the curve [AUC] 0.995, *p* < 0.001) and 11.8 mm for risk of dysplasia or cancer (AUC 0.986, *p* < 0.0001) [53]. These observations align with a large observational study by Szpakowski and Tucker (2020), who followed over 35,000 patients and found that none of the 507 polyps growing to ≥10 mm developed into cancer, implying that growth is a normal biological behavior rather than a predictive sign of malignancy [51]. More recently, Heinrich et al. found that most incidentally detected gallbladder polyps remained stable, regressed, or disappeared during follow-up, with non-negative growth observed in only 20.3% of polyps larger than 6 mm and GBC diagnosed in just one patient [54].

Gallbladder polyps may represent GBC precursors, although genomic studies suggest more complex carcinogenic pathways than a simple adenoma-to-carcinoma sequence [55]. Although CTNNB1 mutations and β-catenin activation may contribute to adenoma/dysplasia-associated GBC, gallbladder carcinogenesis appears genetically heterogeneous rather than driven by a single linear pathway [55,56]. In contrast to colorectal cancer, microsatellite instability appears to play only a minor role in the development of GBC from adenomatous lesions [56].

In conclusion, the surveillance of gallbladder polyps in the prevention of GBC remains challenging given the higher prevalence of benign lesions in the population and low malignant potential, which is ~0.4% even in polyps >10 mm [57,58]. However, this statement should be balanced against the detrimental effects of missed GBC diagnosis. The cornerstone of treatment for gallbladder polyps >10 mm includes prophylactic cholecystectomy, a cut-off that is being questioned given the low incidence of cancer cases that this yields [44,51]. Future preventive strategies should rely on robust risk stratification that combines polyp characteristics, such as size and sessile morphology, with patient-specific factors including age > 60 years, PSC, and Asian ethnicity, thereby distinguishing the small subset of adenomatous polyps with malignant potential from the majority that do not require intensive surveillance or surgical management [45,58]. We also argue that improved imaging techniques such as contrast-enhanced ultrasound, molecular biomarkers, and advanced risk prediction models should be incorporated into these strategies for the appropriate selection of individuals for surveillance and potential treatment.

#### 3.1.4. Primary Sclerosing Cholangitis

Primary sclerosing cholangitis (PSC) is a chronic cholestatic liver disease characterized by intra- and extra-hepatic bile duct injury with a malignant potential [59]. Cholangiocarcinoma is its most characteristic malignant complication and a major determinant of prognosis [60]. Although current literature reports 9 to 78 times higher risk for GBC in patients with PSC, the magnitude of increased risk remains unclear as estimates vary widely in the literature [61].

Previous retrospective studies have reported highly variable estimates of GBC prevalence among patients with PSC, ranging from approximately 2.5% to 21%, with higher proportions reported in selected surgical or transplant cohorts [62]. In a recent meta-analysis, Souza et al. reported 80 GBC cases across 6 PSC studies involving 9440 patients, corresponding to a pooled incidence of 1.06 per 1000 person-years, and 1.52 per 1000 person-years among patients with concomitant inflammatory bowel disease (IBD) [63]. The findings of this meta-analysis suggest low incidence of GBC in PSC patients and implicate that frequent cancer surveillance may not be cost-effective [63].

Polypoid lesions are also relatively common in patients with PSC with prevalence ranging from 10% to 17% [62,64]. A recent systematic review of 6 studies including 3415 PSC patients found gallbladder polyps in 6.2%, of which 16.4% were identified as mass lesions; among those undergoing cholecystectomy, GBC was found in 41.4% of resected polyps, with malignant polyps averaging 2.1–2.4 cm compared to 0.6–1.5 cm for benign lesions [65]. Notably, a case–control study of 363 PSC patients reported that even small polyps can have malignant potential, with adenocarcinoma observed in a 4- and 7 mm polyp; however, in another retrospective study polyps <8 mm showed no significant growth after a median follow-up of 5 years [62,64]. Additionally, IBD appears to confer an additive risk of GBC among patients with PSC, as demonstrated in a propensity score-matched analysis (adjusted hazard ratio, aHR, 4.14; 95% CI: 1.69–10.14) [66].

Similarly to cholangiocarcinoma, the pathogenesis of GBC in PSC patients is primarily mediated by chronic cholestasis-induced inflammation which exposes gallbladder epithelium to high levels of IL-6, TNF-a and toxic bile acids [67]. A histopathologic analysis of gallbladders in PSC patients undergoing liver transplantation demonstrated pyloric metaplasia in 96%, intestinal metaplasia in 50%, dysplasia in 37%, and adenocarcinoma in 14% of surgical specimens which supports a metaplasia-dysplasia-carcinoma sequence [68]. Overall, the toxic environment created by bile stasis along with the contribution of early genetic alterations (i.e., TP53, CDKN2A, and ERBB2) promote an accelerated cascade of carcinogenesis in patients with PSC [59,69].

Although PSC appears to be a more well-established clinical entity associated with GBC, the clinical implications of this association remain debatable. Current evidence has led to conflicting guideline recommendations from the societies with the American Association for the Study of Liver Diseases and European Association for the Study of the Liver recommending cholecystectomy regardless of polyp size in PSC patients, while the American College of Gastroenterology recommends cholecystectomy only for polyps >8 mm, and the American Gastroenterological Association suggests individualized approach based on polyp size, growth, and the patient’s clinical status [61,70,71,72]. Despite the low incidence of GBC in PSC patients, the main argument for continued cancer surveillance includes the high risk of malignancy that the disease confers. There is a growing need for prospective longitudinal studies to validate the clinical utility and cost-effectiveness of annual GBC surveillance in this population.

#### 3.1.5. Chronic Biliary Infection

Chronic biliary infection is a biologically plausible and regionally important pathway in gallbladder carcinogenesis. Among microbial exposures, chronic carriage of *Salmonella enterica* serovars *Typhi* and *Paratyphi* is the most strongly supported determinant, followed by more heterogeneous evidence for *Helicobacter* species [7,11,73,74,75]. The proposed link is persistent colonization of the gallbladder and biliary tract, causing chronic inflammation, epithelial injury, and, in some cases, direct effects on host–cell signaling [73,74,76]. In endemic regions, particularly South and Southeast Asia, a minority of infected individuals develop chronic asymptomatic carriage, creating a reservoir for both transmission and long-term gallbladder exposure [74,77].

The epidemiological signal for *S. Typhi/Paratyphi* is substantial. A landmark cohort study of chronic typhoid and paratyphoid carriers in Scotland reported markedly elevated GBC mortality compared with the general population (RR 167; 95% CI: 54–389), although the estimate was based on few events [78]. Subsequent pooled case–control evidence has supported a more moderate but consistent association, with chronic *S. Typhi* carrier status associated with an approximately four- to five-fold higher risk of GBC [7,75,77,79,80,81,82,83,84,85,86,87]. In a prospective analysis from India among patients with gallstones, chronic *S. Typhi* carriage was independently associated with GBC (OR 14; 95% CI: 2–92), reinforcing the interaction between bacterial persistence and gallstone disease [88].

Evidence for *Helicobacter* species is less specific but remains relevant to biliary carcinogenesis. An umbrella review of 38 meta-analyses reported a positive association between *H. pylori* colonization and biliary tract cancer risk (OR 2.67; 95% CI: 1.57–4.52). CagA positivity was also positively associated with biliary tract cancer, although with a smaller effect estimate (OR 2.19; 95% CI: 1.07–4.50) [89]. In the Finnish Alpha-Tocopherol, Beta-Carotene Cancer Prevention Study, *Helicobacter pylori* seropositivity was associated with biliary tract cancer, but a hospital-based case–control study from India did not find a difference in *H. pylori* serology between GBC cases and controls [90,91]. Non-pylori *Helicobacter* species, particularly *H. bilis* and *H. hepaticus*, may be more directly relevant to biliary tract disease because they have been detected in bile and biliary tissue [92,93,94].

Mechanistically, infection-related gallbladder carcinogenesis likely involves two complementary pathways: sustained inflammation with cytokine and oxidative signaling, and bacterial factors capable of altering host–cell proliferation, apoptosis, differentiation, or DNA integrity [73]. *Salmonella* can persist in gallstones through biofilm formation, creating a protected niche that favors chronic colonization and limits antibiotic eradication [76]. Experimental work further supports causality: chronic *Salmonella* infection has been shown to promote gallbladder carcinogenesis through activation of the epigenetic modulator KDM6B, while *Salmonella* manipulation of host MAPK and AKT signaling can provoke cellular transformation associated with GBC [95,96].

From a prevention perspective, chronic *Salmonella* carriage is one of the few GBC determinants with a direct, infection-targeted intervention pathway. At the global level, *S. Typhi* carriage has been estimated to account for approximately 4.5% of GBC cases (95% UI: 2.2–7.7), but the attributable burden is likely much higher in endemic regions [8]. Prevention should prioritize typhoid vaccination, water, sanitation, and hygiene infrastructure, and detection and treatment of chronic carriers [97]. Because established gallbladder colonization may be protected by biofilms, antibiotic therapy alone may be insufficient; in selected chronic carriers, particularly those with gallstones, elective cholecystectomy may be considered to eliminate the gallbladder reservoir and reduce subsequent GBC risk [76,78,97]. The role of ultrasound surveillance in chronic carriers who do not undergo cholecystectomy remains uncertain and requires prospective evaluation.

#### 3.1.6. Congenital Biliary Diseases

Congenital biliary anomalies, mainly pancreaticobiliary maljunction (PBM) with or without choledochal cysts/congenital biliary dilatation, are recognized risk factors for biliary tract cancers, including GBC [16]. Congenital biliary dilatation is usually associated with PBM, although PBM can also occur without biliary dilatation [16,98,99]. In a Japanese nationwide survey, biliary tract cancer was reported in 22% of adults with PBM and biliary dilatation and in 42% of adults with PBM without biliary dilatation, with GBC predominating in the latter group [16,17].

A systematic review and meta-analysis of 18 observational studies including 2904 patients with choledochal malformations reported an overall biliary malignancy rate of 10.7% [100]. Malignant transformation occurred predominantly in choledochal cysts type I and IV, and biliary cancer was most found in patients with cyst drainage rather than in those who underwent complete cyst excision (OR 3.97, 95% CI: 2.40–6.55) [100]. A large review by Sastry et al. including 78 publications and 5780 patients found that cholangiocarcinoma and GBC were the most common malignancies, with malignancy reported in 11.4% of adults, and with risk increasing with age [101]. Although complete cyst excision reduces malignant risk, residual long-term risk persists, as shown in several patients’ series reporting biliary tract malignancy after excision [102,103,104]. In a Japanese cohort, the cumulative incidence of biliary tract cancer after cyst excision was 1.6%, 3.9%, and 11.3% at 15, 20, and 25 years, respectively [105].

The most recent and robust evidence on the association between PBM and GBC comes from a systematic review and meta-analysis conducted by Rivero-Moreno et al. in 2025 [18]. Among 15 observational studies encompassing 8604 patients, the presence of PBM conferred an 8.42-fold increased risk of biliary cancer (95% CI: 3.57–19.87), with a significantly higher risk for GBC than for bile duct cancer (OR 16.91 vs. 3.36; *p* = 0.003) [18]. The Japanese nationwide survey of patients with PBM reported biliary cancer in 21.6% of adult patients with congenital biliary dilatation and in 42.4% of those without biliary dilatation, with GBC accounting for 62% and 88% of cancers in each group, respectively [19]. Importantly, Muraki et al. identified PBM in approximately 8% of GBC cases across both Western (24/300 U.S. cases) and Eastern (15/171 Japanese cases) cohorts, suggesting that this condition is not limited to Asian populations [106]. In a retrospective Japanese study, PBM was identified in 16% of GBC cases but only 0.44% of patients undergoing cholecystectomy for benign gallbladder disease, supporting a strong association with GBC [107].

PBM and choledochal cysts is thought to induce carcinogenesis through chronic bile stasis with reflux of pancreatic juice causing injury to the biliary epithelium [16]. Activated pancreatic enzymes and secondary bile acids may cause persistent epithelial injury through chronic inflammation and hyperplastic mucosal changes that progress through a hyperplasia–dysplasia–carcinoma sequence [108]. At the molecular level, mutations in the *KRAS* proto-oncogene are more frequently found in cases of PBM compared to patients with carcinogenesis associated with gallstones, in which early inactivation of TP53 is more commonly involved [16,108].

Given the well-established risk of malignant transformation, prophylactic surgical treatment is recommended for all patients with PBM, irrespective of symptoms [109]. For choledochal cysts, complete cyst excision with Roux-en-Y hepaticojejunostomy constitutes the standard therapeutic approach, as internal drainage procedures are linked to a higher risk of subsequent biliary-tract malignancy [100,109]. Additionally, the lifelong higher risk for GBC even after complete excision of the cyst, requires continued surveillance, especially for patients with types I and IV cysts after resection [110]. Given the occurrence of PBM-related GBC at significantly younger ages, the development of non-invasive (e.g., MRCP) strategies for the early detection of PBM without biliary dilatation is essential to reduce the risk of GBC, especially in patients with gallbladder wall thickening on ultrasonography.

### 3.2. Metabolic Health and Adiposity

Beyond gallbladder-specific diseases and congenital abnormalities, adiposity-related measures show the most consistent and robust associations with GBC risk among potentially modifiable exposures [7].

A pooled analysis across 32 prospective cohorts, including over 9 million participants, demonstrated a robust, continuous dose–response relationship between increasing body mass index (BMI) and GBC risk, with a 27% increase in risk per 5 kg/m^2^ increment (RR 1.27; 95% CI: 1.21–1.33) [7,111,112,113,114,115,116]. In a recent evidence synthesis, this continuous BMI association was graded as high credibility, based on criteria including statistical significance, number of incident cases, between-study heterogeneity, small-study effects, and robustness to unmeasured confounding using E-values [7]. The relationship was confirmed across categorical contrasts: overweight (BMI 25–30 kg/m^2^) was associated with a 28% excess risk (RR 1.28; 95% CI: 1.14–1.43; moderate credibility) [111,113,114,117,118,119], and obesity (BMI > 30 kg/m^2^) with a 70% excess risk relative to normal weight (RR 1.70; 95% CI: 1.44–2.01; high credibility) [113,114,117,118,119,120]. A clear gradient was apparent at the extreme end of the spectrum, with class III obesity (BMI > 40 kg/m^2^) associated with a more than three-fold increase in GBC risk (RR 3.32; 95% CI: 2.15–4.80) [113].

Recent consensus has moved away from defining obesity by BMI alone, reconceptualizing it as a chronic disease of excess or dysfunctional adiposity causing organ impairment, with particular emphasis on fat distribution and adipose tissue dysfunction as the biologically relevant drivers [121]. Consistent with this view, measures of central adiposity showed associations comparable to or stronger than BMI-defined obesity, with high-credibility estimates across metrics and cohorts [7]. Waist-to-height ratio was associated with a 49% increase in GBC risk per 0.1-unit increment (RR 1.49; 95% CI: 1.36–1.64; high credibility) [113,115], one of the strongest and most consistent central-adiposity signals identified. Waist circumference showed a 14% increase per 5 cm increment (RR 1.14; 95% CI: 1.10–1.18; high credibility) [113,115], while a categorical high waist circumference was associated with a doubling of risk (RR 2.03; 95% CI: 1.23–3.35; low credibility) from a single pooling project [122]. These findings suggest that carcinogenic risk is driven less by total body mass than by metabolically active adipose tissue distribution, aligning with the framework proposed by Rubino and colleagues [121].

Associations between adiposity and GBC were consistently stronger and more credible in women than in men: the BMI–GBC relationship on the continuous scale reached high credibility in females (RR 1.24; 95% CI: 1.13–1.35) [116,122] but only low credibility in males (RR 1.22; 95% CI: 1.01–1.49) [122]. Similarly, measures of central adiposity, including waist circumference and waist-to-height ratio, reached statistical significance in women but not in men [122]. This pattern is consistent with the recognized female predominance of GBC and with evidence linking adiposity, parity, and gallstone-related pathways to increased risk, supporting a biologically plausible overlap between adiposity-related metabolic dysfunction, hormonal influences, biliary stasis, and gallstone formation [123,124].

Limited evidence suggests that weight gain during adulthood may also increase GBC risk, with a modest elevation per standard deviation of weight gain (RR 1.18; 95% CI: 1.05–1.33; low credibility) [122] and a higher risk among individuals with substantial BMI gain over time (RR 1.77; 95% CI: 1.16–2.70; low credibility) [113]. These findings point to a potential role for sustained adiposity exposure across the life course [7].

The biologic plausibility of these associations is supported by multiple converging mechanisms. Excess and dysfunctional adipose tissue promote systemic inflammation through adipokine dysregulation and kynurenine pathway activation, while associated metabolic disturbances, including insulin resistance and hyperinsulinemia, contribute to gallstone formation and bile stasis, both established precursors of GBC [125,126]. Prospective studies have demonstrated that the effects of adiposity on GBC risk operate independently of gallstone status, supporting a direct carcinogenic pathway beyond the indirect route through cholelithiasis [7,122]. The International Agency for Research on Cancer has classified body fatness as a sufficient cause of GBC, and the framework proposed by Rubino and colleagues reinforces that adipose tissue dysfunction drives organ-level impairment [121,127].

A recent population-level simulation study has estimated that central obesity alone accounted for 29.7% of GBC cases globally (95% uncertainty interval, UI, 8.7–49.5), corresponding to an estimated more than 30 thousand attributable cases annually. This share was higher in women (population attributable fraction, PAF 32.5%; 95% UI: 9.8–52.8) than in men (23.9%; 95% UI: 6.5–41.9), consistent with the sex-specific epidemiological pattern described above [8]. The combined PAF for overweight and obesity was 15.9% (95% UI: 11.6–20.2), corresponding to approximately 20 thousand additional attributable cases per year. Notably, central obesity alone represented the largest single modifiable contributor identified, accounting for nearly one-third of the global GBC burden [8].

Overall, findings across BMI, central adiposity measures, and weight change point in the same direction, suggesting that excess and dysfunctional adiposity is a key modifiable driver of GBC risk [7,8]. Consistently, the prevention implications are largely overlapping, supporting a unified focus on metabolic health and excess adiposity rather than metric-specific thresholds. Global strategies promoting healthy body weight, reduction of abdominal adiposity, and prevention of weight gain across adulthood align with WHO targets for non-communicable disease prevention and, based on the PAF estimates reviewed here, could potentially prevent up to a maximum of one-third of GBC cases worldwide [8]. Emerging metabolic therapies, such as GLP-1 receptor agonists, may offer novel possibilities for risk modification; however, their impact on GBC incidence remains to be established in prospective studies. These findings also provide a mechanistic framework through which other modifiable lifestyle factors, particularly physical activity and sedentary behavior, may influence GBC risk.

### 3.3. Physical Activity and Sedentary Behavior

Meta-analyses of longitudinal studies have shown that high total physical activity was associated with a 45% reduction in GBC risk compared to low activity (RR 0.55; 95% CI: 0.38–0.80) [7,128,129], while high leisure-time physical activity was associated with a 24% reduction (RR 0.76; 95% CI: 0.63–0.91) [7,129,130,131]. Similarly, physically active occupational activity was associated with a lower GBC risk compared to sedentary employment (RR 0.64; 95% CI: 0.46–0.89) [7,129,132]. Notably, the direction of association was consistent across activity domains, despite all estimates being classified as low credibility, reflecting the limited number of available studies and events rather than inconsistency in effect direction. Consistently, low leisure-time physical activity was associated with increased GBC risk (RR 1.32; 95% CI: 1.10–1.58; low credibility) [7,129,130,131]. In contrast, GBC-specific evidence for sedentary behavior remains insufficient, with non-significant associations observed for leisure-time sitting (RR 1.40; 95% CI: 0.86–2.29), limiting firm quantitative conclusions [7,130,133].

The biologic plausibility of a protective role for physical activity in GBC is strong. Epidemiologic evidence supports an inverse association between physical activity and overall cancer risk, and an independent role of sedentary behavior on cancer development [134]. These relationships are mediated through mechanisms that overlap directly with those implicated in GBC carcinogenesis, including improvements in insulin sensitivity, reductions in systemic inflammation, and modulation of immune surveillance [134]. Physical inactivity and prolonged sedentary behavior likely reflect a broader state of metabolic dysfunction, characterized by insulin resistance, low-grade inflammation, and adipose tissue dysregulation, all of which are relevant to GBC pathogenesis [135,136,137]. Large-scale, individual participant data meta-analyses using device-measured activity further demonstrate that even small increases in moderate-to-vigorous physical activity are associated with meaningful reductions in mortality, particularly among the least active individuals, highlighting a steep and non-linear dose–response relationship [138]. Although not specific to GBC, these findings reinforce the concept that modest shifts in population-level behavior can translate into substantial reductions in disease burden.

At the population level, the convergence of GBC-specific epidemiological evidence, mechanistic insights, and broader population data supports a coherent public health strategy. A modelling study has suggested that physical inactivity may account for approximately one-fifth of global GBC cases [8]. Interventions should target multiple domains of activity, including leisure-time, daily movement, and when possible, occupational. Reducing sedentary time represents a complementary actionable strategy. Physical activity promotion is also likely to exert synergistic effects, acting both directly and through improvements in central adiposity and metabolic health, thereby amplifying its overall impact. As physical inactivity continues to rise globally [139], the rationale for integrating activity promotion into GBC prevention strategies becomes increasingly strong.

### 3.4. Other Lifestyle and Socioeconomic Determinants

Other lifestyle and socioeconomic factors represent a heterogeneous group of GBC determinants, ranging from behavioral exposures such as tobacco smoking and alcohol consumption to structural determinants such as educational attainment, each with distinct evidence profiles, effect sizes, and prevention implications. Taken together, these factors account for a meaningful share of global GBC burden [7,8].

#### 3.4.1. Tobacco Smoking

Tobacco smoking has been examined across multiple longitudinal analyses, with approximately half of the available meta-analyses reaching statistical significance [7].

Overall, smoking is associated with a modest increase in GBC risk, with significant but low-credibility associations observed for “ever smoking” (RR 1.15; 95% CI: 1.05–1.25) [7,140,141,142,143,144], current smoking (RR 1.27; 95% CI: 1.02–1.57) [120,142,144,145], and heavy smoking (RR 1.23; 95% CI: 1.12–1.36) [140,142,144,145]. Although several associations reached statistical significance, they showed generally modest magnitude and lacked a clear dose–response pattern, limiting confidence in a causal interpretation [7]. Continuous analyses showed no significant association between smoking pack-years and GBC risk in the largest available pooling project of 26 prospective cohorts, despite dose–response relationships observed for other biliary tract subsites [142]. Importantly, associations observed across other biliary tract cancers, including cholangiocarcinoma and ampullary cancer, do not necessarily apply to the gallbladder, and biliary-tract-wide findings should not be generalized to GBC [145].

Despite this epidemiologic uncertainty, biologic plausibility supports a contributory role. Experimental studies have demonstrated that tobacco-specific nitrosamines such as 4-(methylnitrosamino)-1-(3-pyridyl)-1-butanone (NNK) can undergo hepatic metabolism and biliary excretion, suggesting a potential pathway for direct epithelial exposure within the gallbladder [146]. Assuming a causal role, a modeling study estimated that tobacco smoking was estimated to account for 3.9% of global GBC cases (95% UI: 0.3–7.9) [8].

Nevertheless, given the established carcinogenic properties of tobacco across multiple organ systems and the biological plausibility of biliary exposure, tobacco control remains a relevant component of a broader GBC prevention framework [147].

#### 3.4.2. Alcohol Consumption

Alcohol consumption has been evaluated across multiple meta-analyses and large prospective cohorts, with overall evidence for an association with GBC remaining weak and inconsistent [7]. Pooled estimates comparing alcohol drinkers with non-drinkers did not demonstrate a significant association (RR 0.84; 95% CI: 0.65–1.08) [120,141,148,149], and dose–response analyses showed no meaningful relationship per incremental increase in alcohol intake (RR per 1 drink/day 0.95; 95% CI: 0.90–1.01) [142], indicating the absence of a consistent exposure–response gradient. Consistent with these findings, large pooled prospective analyses have not identified a convincing association between alcohol consumption and GBC risk across intake levels [7].

Some evidence suggests a potential increase in risk at higher levels of exposure; frequent alcohol consumption has been associated with a modest elevation in GBC risk (RR 1.23; 95% CI: 1.04–1.46) [150,151], although this finding is based on a limited number of studies and has been classified as low credibility. Individual cohort studies similarly report increased risks among heavy drinkers, including hazard ratios exceeding three-fold in extreme exposure categories [149,151]. More recent large-scale analyses indicate that the association between alcohol and GBC may be modified by metabolic context, with stronger effects observed in individuals with prediabetes or diabetes and evidence of synergistic interactions between alcohol exposure and metabolic dysfunction [150]. However, these findings are not consistent across studies and do not establish a clear dose–response pattern at the population level.

Despite the lack of consistent epidemiologic evidence, biologic plausibility for a contributory role of alcohol in GBC carcinogenesis exists. Ethanol is metabolized to acetaldehyde, a recognized Group 1 carcinogen, which can induce DNA damage and interfere with DNA repair processes [147]. Chronic alcohol exposure promotes oxidative stress and inflammatory signaling pathways implicated in carcinogenesis [152]. Although gallstones and chronic gallbladder inflammation are central pathways in GBC development, this mechanism is unlikely to explain any alcohol-GBC association. In contrast, alcohol consumption has been inversely associated with gallstone disease in dose–response meta-analyses, with proposed mechanisms including reduced bile cholesterol saturation and favorable HDL-related lipid changes [153,154,155,156,157]. Therefore, if alcohol contributes to GBC risk, this effect is unlikely to be mediated primarily through promotion of gallstone disease and may instead reflect direct carcinogenic mechanisms such as acetaldehyde-related DNA damage or oxidative stress.

Despite these conflicting results, assuming a detrimental causal role, alcohol consumption has been estimated to account for 7.0% of global GBC cases (95% UI: 1.2–13.0) [8]. However, this estimate should be interpreted cautiously considering the inconsistent epidemiologic evidence. The apparent discrepancy between weak individual-level associations and a non-trivial PAF is largely explained by the high prevalence of exposure, and warrants continued monitoring even in the absence of a confirmed causal relationship.

#### 3.4.3. Educational Attainment

High educational attainment represents one of the most robust protective associations identified across modifiable exposures in a recent evidence synthesis, with high-credibility evidence observed in sex-stratified analyses [7]. Compared with low educational attainment, higher education was associated with a substantially reduced risk of GBC (RR 0.63; 95% CI: 0.49–0.82) [119,141], with moderate overall credibility across four studies including 122,029 participants and 1310 cases. This association was stronger and reached high credibility in women (RR 0.55; 95% CI: 0.44–0.70) [119,141], whereas the corresponding estimate in men was not statistically significant (RR 0.76; 95% CI: 0.53–1.09) [119,141]. The more pronounced association observed in women may partly reflect confounding or mediation by reproductive factors such as parity, an established risk factor for GBC that is inversely associated with educational attainment in many populations [141,158]. However, this does not negate the protective association of education itself, but instead suggests that socioeconomic and reproductive pathways may converge to amplify risk in female populations.

Beyond GBC-specific evidence, educational attainment has been consistently associated with reduced all-cause and cancer-specific mortality in a dose–response manner across diverse populations [7]. Large-scale global analyses by the Institute for Health Metrics and Evaluation-Centre for Global Health Inequalities Research (IHME-CHAIN) Collaborators demonstrated that increasing levels of education are associated with progressively reduced adult mortality risk across all world regions [159], while earlier studies have shown persistent educational inequalities in cause-specific mortality across European populations [160].

Despite this evidence, education is not thought to be a direct biological exposure but a structural determinant operating through multiple downstream pathways. Lower educational attainment likely captures a broader socioeconomic gradient encompassing poorer dietary quality, higher prevalence of unhealthy behaviors, greater occupational exposures, and reduced access to and engagement with healthcare systems [8,161]. This is further supported by observations of an inverse relationship between cholecystectomy rates for cholelithiasis and GBC incidence, suggesting that delayed access to surgical care in socioeconomically disadvantaged populations may contribute to sustained exposure to gallstone-related inflammation [162]. These pathways converge on metabolic dysfunction, chronic inflammation, and delayed disease detection, mechanisms central to gallbladder carcinogenesis, linking socioeconomic disadvantage to biologic risk [163].

From a population perspective, low educational attainment accounted for an estimated 9.1% of global GBC cases (95% UI: 3.7–15.1) [8]. Despite its indirect mode of action, this places education among the leading modifiable contributors to global GBC burden. The magnitude of this estimate is notable given that education exerts its effects through multiple intermediate exposures, suggesting that its population-level impact may extend beyond what is captured by single-exposure attributable fraction models.

From a prevention perspective, improving educational attainment may reduce GBC risk through its influence on several established risk factors, including obesity, smoking, physical inactivity, and access to healthcare. Unlike interventions targeting individual behaviors alone, these findings suggest that broader social and educational policies may also contribute to GBC prevention, particularly in high-incidence populations where socioeconomic disadvantages and metabolic risk factors frequently coexist.

### 3.5. Reproductive and Hormonal Determinants

Reproductive and hormonal factors have long been considered relevant to GBC, given the marked female predominance of the disease and the established role of estrogen in promoting biliary cholesterol secretion and cholelithiasis. In a recent evidence synthesis, however, only parity emerged as a consistent determinant on longitudinal evidence, while exogenous hormone exposures showed no reliable association [7].

Higher parity is associated with a modest increase in GBC risk, with high parity carrying a 48% excess risk relative to low parity (RR 1.48; 95% CI: 1.30–1.68; moderate credibility) [158,164,165]. A continuous analysis from the Biliary Tract Cancers Pooling Project similarly indicated a 7% increase in risk per additional birth (RR 1.07; 95% CI: 1.03–1.11) [158]; this estimate was derived from a single pooling project in which heterogeneity and small-study effects could not be assessed, and the credibility grade should therefore be interpreted with caution. The association is biologically coherent: pregnancy raises circulating estrogen and progesterone levels, increasing biliary cholesterol saturation and gallbladder stasis, both of which promote gallstone formation and chronic inflammation, the principal precursor pathway for GBC [7,166]. The lithogenic effect of pregnancy is well characterized: biliary sludge and gallstones arise in up to 30% and 12% of women respectively during pregnancy and the postpartum period, driven by estrogen-induced cholesterol supersaturation of bile and reduced gallbladder motility [166]. In principle, this identifies a mechanistic point of intervention, pharmacological strategies that counter gestational cholelithogenesis, such as ursodeoxycholic acid or lipid-lowering agents, could theoretically interrupt the gallstone-mediated pathway to GBC [167]. In practice, however, such an approach would be disproportionate to the relatively small absolute increase in GBC risk associated with parity, would raise clear safety concerns during pregnancy, and cannot be regarded as a realistic prevention strategy; it is noted here only to illustrate the biological continuity from reproductive hormones through gallstone formation to carcinogenesis. Furthermore, parity is strongly associated with other factors, such as educational attainment and other socio-economic factors, with important heterogeneity around the globe. Hence, the potentially complex relationship of this factor with other more credible ones, makes positioning its exact role in prevention even more controversial [8].

By contrast, exogenous hormone exposures showed no convincing association with GBC in longitudinal data. Ever-use of exogenous female hormones (RR 1.43; 95% CI: 0.86–2.39) [148,168], oral contraceptives (RR 1.32; 95% CI: 0.42–4.21) [169,170], and hormone replacement therapy (RR 1.18; 95% CI: 0.57–2.45) [171,172] were not statistically significant. However, these null findings should be interpreted cautiously, as the available evidence was based on relatively few studies and limited case numbers, resulting in wide confidence intervals. Measures of reproductive timing, including nulliparity and age at first birth, were likewise null [7].

From a prevention standpoint, these factors do not represent direct preventive measures and are best suited as possible markers for risk stratification of higher-risk women, particularly in high-incidence regions. Reproductive factors were not among the exposures included in the global attributable-burden analysis [8], consistent with their limited or controversial role in primary prevention.

### 3.6. Dietary Patterns and Micronutrients

Diet has been widely studied in relation to GBC, especially in high-incidence regions of South Asia, but remains one of the least secure exposure domains. Most reported associations come from small retrospective case–control studies, whereas longitudinal evidence has not identified any dietary factor with sufficient consistency, temporality, or credibility to support a causal interpretation [7]. The main limitations are heterogeneous dietary definitions, recall and selection bias, residual confounding, and extensive testing of individual foods and nutrients.

The literature nevertheless contains recurrent signals. Vegetarian or plant-rich dietary patterns, fruits, selected vegetables and plant foods (including potato, onion and garlic, aubergine, cabbage, carrot, and mustard leaves) and higher zinc or selenium concentrations in serum or bile have been reported as inversely associated with GBC risk [7]. Accordingly, some studies have linked non-vegetarian or region-specific dietary patterns with higher risk of the disease [173,174,175,176]. These findings are etiologically interesting but not yet actionable. Dietary categories may be acting primarily as markers of socioeconomic position, gallstone disease, metabolic health, infection risk, water quality, cooking practices, food storage, or environmental exposures, rather than as independent causal factors.

This uncertainty is illustrated by a northern Indian gallstone-matched case–control study using a detailed questionnaire with 105 food items: non-vegetarian diet, coffee, and sweets emerged as candidate factors, but in poorly powered multivariable models, high coffee and sweets intake appeared inversely rather than positively associated with GBC risk [176].

Biologically, diet could influence GBC through gallstone formation, bile cholesterol saturation, adiposity, insulin resistance, systemic inflammation, bowel habits, and foodborne contaminants. However, no specific food, nutrient, or dietary pattern can currently be considered an established modifiable determinant of GBC. Diet was also not included as a separate exposure in the global attributable-burden analysis [8], reflecting the absence of robust causal estimates. Therefore, dietary patterns may contribute to GBC prevention mainly through their effects on adiposity, metabolic dysfunction, gallstone disease, and unsafe food-related exposures, rather than through prescription or elimination of single foods.

### 3.7. Toxicants and Environmental Exposures

Environmental and occupational exposures have been investigated as GBC determinants, particularly in high-incidence regions where specific contaminants are prevalent. As with diet, most of this evidence derives from case–control studies; two exposures, however, warrant specific attention because of their biological plausibility and, in the case of aflatoxin, the existence of a clear prevention pathway.

#### 3.7.1. Aflatoxin

Aflatoxins are potent hepatocarcinogenic mycotoxins produced by *Aspergillus* species contaminating improperly stored grains, nuts, and maize, particularly in warm, humid climates [177]. Evidence linking aflatoxin exposure to GBC comes from case–control studies using biomarkers of internal dose: pooled analysis showed that detectable aflatoxin B1-lysine adducts in plasma were associated with a more than four-fold increased risk of GBC (RR 4.33; 95% CI: 1.33–14.1) [178,179]. The association is biologically well grounded: aflatoxin B1 is a recognized Group 1 human carcinogen that, following metabolic activation, forms DNA adducts and induces characteristic mutations, with studies in high-incidence GBC populations in Chile and China implicating biliary aflatoxin exposure specifically [177,180]. Unlike most GBC determinants, aflatoxin exposure is directly preventable through measures that reduce contamination of the food supply: improved crop drying and storage, regulatory limits on aflatoxin concentrations in food, and agricultural biocontrol using atoxigenic *Aspergillus flavus* strains to reduce contamination before and after harvest. In high-incidence, high-exposure regions, these represent a concrete and biologically justified prevention strategy [181,182].

#### 3.7.2. Drinking-Water Contamination (Heavy Metals)

Evidence on toxic metals and drinking-water contamination remains limited but biologically and geographically relevant. A recent evidence synthesis identified several contaminants, including arsenic, cadmium, chromium, lead, boron, lithium, molybdenum, and pesticide compounds, as signals requiring further longitudinal investigation [7]. Among these, the most directly prevention-relevant evidence concerns arsenic-contaminated drinking water: in a case–control study from arsenic-impacted regions of India, long-term average-weighted groundwater arsenic exposure was associated with higher GBC risk in a dose–response pattern, with ORs of 2.00 and 2.43 across increasing tertiles of exposure [183]. A plausible exposure source is contaminated drinking water in regions affected by industrial pollution or naturally elevated groundwater metal concentrations; biliary excretion and accumulation in bile or gallbladder tissue have been proposed as potential routes of gallbladder exposure [183,184]. Additional biomarker-based studies from northern India and China have reported associations between GBC and elevated concentrations of metals such as cadmium, chromium, copper, nickel, and arsenic in serum or bile [185,186]. However, these studies are generally small, often case–control or cross-sectional in design, and susceptible to reverse causation because malignancy itself may alter trace-metal handling. Therefore, the evidence should be regarded as regionally suggestive rather than established, although prevention through reduction of arsenic and toxic-metal exposure in drinking water remains justified on broader public-health grounds.

## 4. From Risk Factors to Prevention: A Pragmatic Framework

A prevention framework for GBC must separate three concepts that are often conflated: etiologic credibility, population impact, and actionability. A determinant with a high relative risk may be too rare for population prevention, whereas a modest determinant may matter if it is common, preventable, and embedded in broader non-communicable disease policy. Conversely, clinical interventions such as cholecystectomy require a much higher threshold of absolute risk than lifestyle or environmental interventions because their harm, costs, and opportunity costs are immediate. These dimensions should not be interpreted interchangeably. An epidemiological association, even when statistically robust, does not by itself establish causality or demonstrate that modifying the exposure will reduce GBC incidence. Similarly, a population-attributable fraction is a model-based estimate that depends on assumptions about causality, exposure distribution, and the counterfactual scenario; it should not be interpreted directly as the proportion of cases that would necessarily be prevented by an intervention. Preventive recommendations therefore require consideration not only of association strength or estimated population burden, but also of intervention efficacy, potential harms, feasibility, and baseline risk. PAFs for individual determinants should also not be interpreted as additive, because several exposures are correlated or may operate through overlapping or mediated pathways. For example, educational attainment may capture differences in adiposity, smoking, reproductive factors, environmental exposures, and access to healthcare; adiposity may influence GBC risk partly through gallstone formation; chronic *Salmonella* carriage is closely linked to gallstones; and associations with alcohol may differ according to underlying metabolic status. Consequently, the combined population impact of multiple determinants cannot be estimated by simply summing exposure-specific PAFs, and future prevention models should explicitly consider mediation, interaction, and clustering of risk factors.

Operationally, Table 1 applies these dimensions by indicating for each determinant the strength and limitations of the evidence, its clinical or public-health actionability, the relevant target population, and whether available evidence supports preventive intervention or primarily informs risk stratification and surveillance. At the population level, potentially actionable prevention targets include metabolic health, physical activity, tobacco control, safer food storage, clean water, sanitation, and social policies that reduce structural risk (Table 1). The strength of GBC-specific evidence varies across these measures, but many are independently supported by their broader benefits for cancer, cardiometabolic, and public-health prevention [8,121,134,139,147]. Their limitation is specificity: they reduce background risk rather than identifying who will develop GBC.

At the clinical level, the priority is to identify the subset of patients in whom risk is high enough to justify intervention. This applies to selected patients with gallstones in high-incidence regions, suspicious gallbladder polyps, porcelain gallbladder with concerning features, PSC-associated gallbladder lesions, and congenital anomalies such as pancreaticobiliary maljunction or choledochal cysts [32,34,45,61,100,109]. Here the relevant question is not simply whether an association exists, but whether potential cancer-prevention benefit is likely to outweigh surgical risk and health-system burden.

In endemic settings, prevention becomes more targeted. Chronic *Salmonella* carriage, aflatoxin exposure, and arsenic-contaminated drinking water are not universal GBC determinants, but they may represent actionable targets where exposure and baseline risk are high [76,78,97,178,179,183]. This argues for geographically tailored prevention interventions rather than a single global strategy. For surgical and surveillance strategies, this balance should also account for operative morbidity, overdiagnosis and unnecessary procedures, competing risks, healthcare-system capacity, cost-effectiveness, and equitable access, particularly when interventions are applied to otherwise asymptomatic populations.

## 5. Conclusions and Future Directions

GBC prevention is unlikely to be solved by a single screening test, biomarker, or lifestyle prescription. The disease arises from the convergence of gallbladder injury, metabolic dysfunction, infection, environmental exposures, ancestry, and social context. Prevention should therefore be risk-adapted: population-wide when interventions are safe and broadly beneficial, clinically targeted when surgery or surveillance may be justified, and geographically focused when infection or toxic exposures contribute to local burden.

The most immediate opportunities are risk-stratified management of high-risk gallbladder conditions, prevention of excess and dysfunctional adiposity, promotion of physical activity, control of chronic *Salmonella* carriage in endemic settings, and reduction of aflatoxin and arsenic exposure where these overlap with high GBC incidence. Other factors, including diet, reproductive history, smoking, alcohol, and socioeconomic disadvantage, remain relevant but differ in interpretation: some are indirect prevention targets, some are markers of baseline risk, and some are best justified by broader cancer- or cardiometabolic-prevention benefits rather than by strong GBC-specific evidence alone.

Future research should address both etiological uncertainty and prevention implementation. Many reported associations remain limited by retrospective designs, small samples, heterogeneous exposure definitions, incomplete control for gallstones and socioeconomic factors, and sparse longitudinal data [7]. Prospective studies in high-incidence regions are needed to clarify the role of diet, micronutrients, contaminants, occupational exposures, non-*Salmonella* infections, smoking, alcohol, and reproductive factors, while carefully distinguishing GBC from other biliary tract cancers and examining variation by sex, ancestry, geography, and baseline gallbladder disease.

For determinants with stronger evidence or clearer actionability, priorities include development and external validation of locally calibrated multifactorial risk-prediction models, prospective evaluation of risk-based screening or surveillance strategies, estimates of NNT and surgical harm, cost-effectiveness analyses of prophylactic cholecystectomy in high-risk groups, improved strategies for identifying and managing chronic *Salmonella* carriers, and better measurement of food- and water-borne contaminants [7,8]. These steps are needed to determine which associations are causal, which are clinically useful for risk stratification, and which interventions provide net benefit in specific populations and healthcare settings.

## Figures and Tables

**Table 1:** Prevention-oriented summary of major determinants of gallbladder cancer.

Determinant	Evidence Strength and Quantitative Impact, Where Available	Main Limitations	Role in Prevention/Risk Management	Target Population	Main Preventive Actions
**Gallstones (Cholelithiasis)**	Strong association in observational studies; causal interpretation supported by MR analysis. However, absolute risk remains low [6,10,20,21,22,23,25,26]. Modelled PAF: 27.9% (95% UI 16.8–40.7) globally [8]. Estimates of the number of cholecystectomies needed to prevent one GBC case were 67 in an Indian study and 80 using a risk-prediction approach in Chile [32,34].	Low absolute risk; routine surgery is not universally required. Size acts as a surrogate for long-standing chronic inflammation.	Clinical risk-stratified intervention	Gallstone patients in high-incidence countries (Chile/India); specific high-risk demographics (women aged 35–49 in endemic settings); eligible individuals selected via appropriate risk stratification.	Consider risk-stratified cholecystectomy in selected high-risk populations rather than routine surgery. Consider risk-stratified cholecystectomy in selected high-risk women in settings. Validated multivariable risk models may help identify groups with a more favorable benefit–harm balance.
**Porcelain Gallbladder and Chronic Inflammation**	Low-quality retrospective evidence; traditionally cited as high risk, but recent cohort studies show much lower risk [36,37,38,39,43]. No reliable PAF or NNT available.	True GBC incidence is lower than historically assumed. The primary driver is chronic inflammation/regeneration, not the calcification itself.	Clinical risk-stratified intervention	Healthy individuals with porcelain gallbladder/calcification; frail/elderly cohorts or those with significant comorbidities; cases with high-risk calcification patterns or symptoms of malignancy	Consider cholecystectomy in selected surgically fit patients; observation may be appropriate in older or frail patients after individualized assessment. Incorporate symptoms, imaging findings, calcification patterns, and additional factors into the evaluation.
**Gallbladder polyps**	Variable evidence based on size; risk is predominantly restricted to true adenomatous polyps ≥ 10 mm [44,45,52]. No reliable PAF or NNT available.	Most polyps are benign (cholesterol/inflammatory). Polyp growth as an isolated predictor of malignancy remains heavily debated. Adenomyomatosis is generally benign and non-neoplastic, although the segmental type has been associated with GBC in and wall thickening may complicate detection of coexisting early GBC. Data are mostly retrospective.	Guideline-based clinical management	Asymptomatic individuals with incidentally detected gallbladder polyps; patients with high-risk features (size ≥ 10 mm, sessile morphology, or growth); high-risk subgroups (age > 60, PSC, or Asian/Indian ethnicity).	Manage according to guideline-based thresholds, considering polyp size together with additional risk factors such as sessile morphology, age, PSC, and growth during follow-up. Develop robust risk stratification combining specific polyp traits with patient factors. Use additional imaging selectively when conventional imaging remains indeterminate.
**Primary Sclerosing Cholangitis (PSC)**	Consistently associated with risk increase; risk is further amplified by concomitant IBD [61,62,63,65,66]. No reliable PAF or NNT available.	Low absolute incidence questions the cost-effectiveness of frequent surveillance. Society guidelines present conflicting dimensional thresholds.	Guideline-based clinical management	Patients diagnosed with PSC, particularly those with concomitant IBD.	Follow society-specific guidance for gallbladder polyps in PSC; recommendations differ regarding size thresholds and should be interpreted in the context of surgical risk and other clinical features.
**Chronic Biliary Infection**	Consistent observational association for chronic S. Typhi carriage. Evidence for *Helicobacter* species is suggestive but requires further validation [7,73,74,76,77,79,80,81,82,83,84,85,86,87,89,90,91,92,93,94]. No reliable global PAF or intervention NNT available.	Historical cohorts were small; pooled observational evidence supports an approximately 4- to 5-fold association. *Helicobacter* data are inconsistent across different regional studies. Antibiotics often fail due to *Salmonella* biofilms on gallstones; utility of routine ultrasound surveillance for non-operated carriers remains unproven.	Endemic-area intervention	Chronic asymptomatic *S. Typhi/Paratyphi* carriers; endemic populations exposed to biliary *Helicobacter* colonization.	Introduce typhoid vaccination, water, sanitation, and hygiene infrastructure. Prevent typhoid infection through vaccination, water, sanitation, and hygiene measures, and identify and manage chronic carriers in endemic settings. Consider cholecystectomy in selected chronic carriers with gallstones when clinically appropriate.
**Congenital Biliary Diseases (PBM and Cysts)**	Substantially elevated risk of biliary malignancy has been reported; specifically associated with Pancreaticobiliary Maljunction (PBM) even without biliary dilatation [16,18,100,106,107]. No reliable global PAF or intervention NNT available.	Carcinogenesis involves distinct KRAS mutations. Malignancy risk increases with age, and a significant residual risk persists decades after complete cyst excision. Data relies on retrospective registries and surveys subject to regional reporting variations.	Guideline-based clinical management	Individuals with PBM or choledochal cysts; adults with PBM without biliary dilatation.	Differentiate management based on dilatation status. Complete cyst excision with biliary reconstruction is the standard management for appropriate choledochal cysts. Consider MRCP when PBM is suspected or ultrasonographic findings remain unexplained.
**Metabolic Health and Adiposity**	High-credibility longitudinal association; central adiposity had a modelled PAF of approximately 30% under the assumptions of the attributable-burden analysis [7,8,111,116,121]. Central obesity: modelled PAF 29.7% (95% UI 8.7–49.5); overweight/obesity: 15.9% (95% UI 11.6–20.2) [8].	Central adiposity was estimated to account for approximately 30% of global GBC cases in a modelling analysis. Excess fat promotes carcinogenesis directly via metabolic pathways, persisting even without gallstones. This risk multiplies at the extreme weight spectrum, where class III obesity (BMI > 40 kg/m^2^) was associated with a >3-fold risk increase.	Population-level prevention	Overweight and obese individuals; women with metabolic dysfunction or excess adiposity; individuals experiencing rapid weight gain.	Promote healthy weight, reduction of central adiposity and prevention of weight gain.
**Physical Activity and Sedentary Behaviour**	Low-credibility evidence due to low event counts but shows a broadly consistent inverse direction across activity domains [7,8,128,129,130,131,132,134,138]. Physical inactivity: modelled PAF 20.5% (95% UI 7.4–34.1) [8].	Low credibility reflects small absolute numbers of cancer cases in prospective cohorts, not data contradictions. Evidence specific to sedentary behaviour remains insufficient, limiting quantitative conclusions.	Population-level prevention	Sedentary individuals and those with low levels of leisure-time or physically active employment; populations at high risk for metabolic dysfunction, low-grade systemic inflammation, and insulin resistance.	Promote physical activity and reduce sedentary behaviour for their established broader health benefits; a GBC-specific preventive effect has not been demonstrated in intervention studies.
**Tobacco smoking**	Low-credibility evidence; shows a modest risk increase but lacks a clear dose–response pattern [7,8,140,141]. Modelled PAF 3.9% (95% UI 0.3–7.9), conditional on a causal association [8].	Weak association compared to other tobacco-related cancers. No robust dose–response relationship has been clearly established for GBC.	Population-level prevention	Current, heavy, or long-term smokers.	Although a causal role in GBC etiology remains uncertain, smoking cessation strategies may be considered within broader cancer-prevention strategies.
**Alcohol Consumption**	Weak and inconsistent data (classified as low-credibility); some studies suggest increased risk at higher levels of consumption or in metabolically vulnerable subgroups [7,8,149,150,151]. Modelled PAF 7.0% (95% UI 1.2–13.0), conditional on a detrimental causal association; interpretation is particularly uncertain given the inconsistent epidemiological evidence [8].	No clear association with GBC. Observed associations may be influenced by co-existing metabolic dysfunction or other confounding factors. No dose–response relationship has been observed.	Population-level prevention	Heavy or frequent alcohol drinkers; individuals with overlapping metabolic conditions.	Limit alcohol intake as part of generic multi-cancer counselling. Monitor alcohol consumption especially in patients with underlying metabolic syndrome.
**Educational Attainment**	High-credibility inverse association, particularly in women; may reflect multiple upstream socioeconomic and healthcare-related pathways [7,8,119,141,159]. Low educational attainment: modelled PAF 9.1% (95% UI 3.7–15.1) [8].	Education is not a direct biologic cause but acts as a proxy for clustered risk factors (poor diet, high parity, environmental/occupational exposures). Low educational attainment is linked with substantial attributable burden but acts mainly through downstream socioeconomic pathways.	Population-level prevention	Socioeconomically disadvantaged populations; low-education women exposed to overlapping risk factors.	Address structural social inequalities and improve equitable access to prevention and healthcare. Ensure timely access to cholecystectomy in disadvantaged regions.
**Reproductive and Hormonal Determinants**	Moderate-credibility evidence for parity (modest risk increase); no convincing evidence for the use of exogenous hormones [7,148,158,164,165,166,167,168,169,170,171,172]. No sufficiently robust PAF or intervention NNT for use in prevention.	The parity-GBC link is confounded by low education and socioeconomic factors, making it hard to isolate as an independent biological cause. Large-scale data shows no statistically significant link between oral contraceptives or HRT and GBC.	Risk stratification	Multiparous women, particularly in high-incidence regions where high parity overlaps with low educational or socioeconomic status.	No GBC-specific preventive intervention is currently established. Reproductive history may contribute to contextual risk assessment in high-incidence populations.
**Dietary patterns and micronutrients**	Very low-credibility evidence; data is limited to retrospective case–control studies with high risk of residual confounding [7,8,173,174,175,176]. No reliable PAF available.	Evidence is limited by recall and selection biases in retrospective designs. Weak signals likely reflect structural confounding factors (e.g., cooking methods, water quality, food refrigeration, or healthcare access) rather than true, independent biological causes.	Limited direct actionability	Individuals in high-incidence regions with poor metabolic health or suboptimal socioeconomic conditions, poor water quality, or unsafe food storage practices.	Targeted or restrictive dietary interventions are not justified for GBC prevention. Focus counseling on indirect drivers (weight management). Enforce water and food safety in endemic zones. No GBC-specific dietary recommendation can currently be made beyond general healthy-diet and metabolic-health guidance.
**Aflatoxin**	Suggestive biomarker-based case–control evidence in high-exposure regions, supported by biological plausibility [177,178,179,180]. No reliable PAF available.	Human evidence relies almost entirely on case–control studies rather than prospective cohorts. Wide confidence intervals reflect low statistical precision.	Endemic-area intervention	Rural communities in warm, humid climates relying on stored staple crops (grains, nuts, maize).	Improve strict regulatory ceilings on aflatoxin in food supplies. Implement biocontrol methods and improved post-harvest drying technologies.
**Drinking-water Contamination (Heavy Metals)**	Regionally specific evidence; a dose–response signal for arsenic has been reported in one endemic-region case–control study [7,183,185]. No reliable PAF available.	Evidence relies on small-scale regional studies lacking long-term prospective follow-up. Data is highly localized and cannot be generalized globally.	Endemic-area intervention	Populations living near industrial pollution centers or in regions with naturally elevated groundwater heavy metal levels.	Provide safe, filtered drinking water in industrial or naturally exposed zones are reasonable actions; however, the GBC-specific preventive effect of these interventions has not been directly evaluated. Monitor groundwater for toxic heavy metals, specifically arsenic, cadmium, and chromium.

Abbreviations: BMI, body mass index; GBC, gallbladder cancer; HRT, hormone replacement therapy; IBD, inflammatory bowel disease; MR, Mendelian randomization; MRCP, magnetic resonance cholangiopancreatography; NNT, number needed to treat; PAF, population attributable fraction; PBM, pancreaticobiliary maljunction; PSC, primary sclerosing cholangitis; UI, uncertainty interval.

## Data Availability

No new data were created or analyzed in this study. Data sharing is not applicable to this article.
